# Characterisation of Films Based on Exopolysaccharides from *Alteromonas* Strains Isolated from French Polynesia Marine Environments

**DOI:** 10.3390/polym14204442

**Published:** 2022-10-20

**Authors:** Patrícia Concórdio-Reis, João R. Pereira, Vítor D. Alves, Ana R. Nabais, Luísa A. Neves, Ana C. Marques, Elvira Fortunato, Xavier Moppert, Jean Guézennec, Maria A.M. Reis, Filomena Freitas

**Affiliations:** 1 Associate Laboratory i4HB—Institute for Health and Bioeconomy, NOVA School of Science and Technology, NOVA University of Lisbon, 2829-516 Caparica, Portugal; 2 UCIBIO—Applied Molecular Biosciences Unit, Department of Chemistry, NOVA School of Science and Technology, NOVA University of Lisbon, 2829-516 Caparica, Portugal; 3 LEAF—Linking Landscape, Environment, Agriculture and Food, Associated Laboratory TERRA, Instituto Superior de Agronomia, Universidade de Lisboa, 1349-017 Lisboa, Portugal; 4 LAQV-REQUIMTE, Department of Chemistry, NOVA School of Science and Technology, FCT NOVA, Universidade NOVA de Lisboa, 2829-516 Caparica, Portugal; 5 CENIMAT|i3N, Department of Materials Science, School of Science and Technology, NOVA University Lisbon and CEMOP/UNINOVA, 2829-516 Caparica, Portugal; 6 Pacific Biotech BP 140 289, Arue Tahiti 98 701, French Polynesia; 7 AiMB (Advices in Marine Biotechnology), 17 Rue d’Ouessant, 29280 Plouzané, France

**Keywords:** *Alteromonas* sp., marine bacteria, exopolysaccharide (EPS), films

## Abstract

This work assessed the film-forming capacity of exopolysaccharides (EPS) produced by six *Alteromonas* strains recently isolated from different marine environments in French Polynesia atolls. The films were transparent and resulted in small colour alterations when applied over a coloured surface (Δ*E_ab_* below 12.6 in the five different colours tested). Moreover, scanning electron microscopy showed that the EPS films were dense and compact, with a smooth surface. High water vapour permeabilities were observed (2.7–6.1 × 10^−11^ mol m^−1^ s^−1^ Pa^−1^), which are characteristic of hydrophilic polysaccharide films. The films were also characterised in terms of barrier properties to oxygen and carbon dioxide. Interestingly, different behaviours in terms of their mechanical properties under tensile tests were observed: three of the EPS films were ductile with high elongation at break (ε) (35.6–47.0%), low tensile strength at break (Ꞇ) (4.55–11.7 MPa) and low Young’s modulus (εm) (10–93 MPa), whereas the other three were stiffer and more resistant with a higher Ꞇ (16.6–23.6 MPa), lower ε (2.80–5.58%), and higher εm (597–1100 MPa). These properties demonstrate the potential of *Alteromonas* sp. EPS films to be applied in different areas such as biomedicine, pharmaceuticals, or food packaging.

## 1. Introduction

Petrochemical-based plastics have widespread use in nowadays society due to their low-cost and useful characteristics, that include good mechanical and thermal properties, heat moulding capacity, suitable gas barrier properties, and high transparency [1,2]. However, due to their limited biodegradability which represents a serious environmental and human health threat, those synthetic plastics are expected to be replaced by novel biodegradable materials [3]. Biopolymers, such as polysaccharides, are a promising substitute since they are biodegradable, biocompatible, and non-toxic, since they are obtained from renewable resources [1,4,5,6]. Applications of polysaccharide-based films include food packaging [3], drug delivery systems [7,8], coatings for medical devices [9], and wound dressings [4,10]. Most such applications have been investigated using polysaccharides extracted from plants (e.g., starch), algae (e.g., alginate), or animal resources (e.g., chitosan). Only recently have microbial polysaccharides, due to their improved properties, started to arise as competitive alternatives to other natural polymers as well as synthetic products [11].

Polysaccharides-based films are considered effective barriers against gases, including oxygen and carbon dioxide, when exposed to low relative humidity conditions [4,12]. This property is relevant for maintaining the food’s nutritional and sensory properties in food packaging applications [3] and the stability of bioactive molecules in drug delivery systems [7]. However, their hydrophilic nature renders them poor barrier properties against water vapour, which limits their application as water barriers in food packaging [3]. On the other hand, they constitute interesting materials to produce film pads with good water absorption capacity to be used in active packages [4]. In addition, due to their hydrophilicity, polysaccharides can form non-covalent bonds with growth factors to support bioadhesion [10] and can absorb the exudate produced during the wound healing process, which are important aspects for wound dressing materials [4,13]. However, to meet the requirements of most applications, the mechanical properties of polysaccharide-based films still need to be improved [3]. To this end, several strategies have been studied, including blending with other polymers, the addition of hydrophobic materials and plasticisers, or chemical modifications of the film-forming polysaccharide [3]. Another strategy is to look for novel polymers with different molecular structures and functional properties that might result in improved films’ properties.

In this context, the potential of microbial polysaccharides is limitless as newly isolated exopolysaccharide-producing bacteria can be considered as sources of polysaccharides with novel or improved properties [11,14,15]. In addition, marine environments, which represent the largest ecosystem of the planet, are an underexplored source of novel microorganisms characterised by high ecological and metabolic diversity with great biodiscovery potential [14]. Exopolysaccharides (EPSs) from marine bacteria display great structural and compositional diversity, which might result in films with improved characteristics. Additionally, marine-derived EPS compositions often include the presence of atypical sugar monomers (e.g., fucose, fructose, rhamnose) and high contents in uronic acids and sulphate groups, which are frequently found in marine-derived EPS, and might confer novel or improved biological activities to these molecules [14,16].

Recently, EPSs from six different *Alteromonas* strains were isolated from unique French Polynesia marine environments, namely Moorea Island lagoon, Fakarava atoll, and Tikehau atoll [14]. The six EPSs are high-molecular-weight heteropolysaccharides composed of neutral monosaccharides (glucose, galactose, mannose, rhamnose, and fucose), and with a high content of uronic acids (glucuronic acid and galacturonic acid) that contribute to their anionic character [14]. The EPS solutions revealed an interesting shear thinning behaviour and could form gels by the addition of Fe^2+^ or in the presence of Mg^2+^, Cu^2+^, or Ca^2+^ under alkaline conditions, which supports their potential as biomaterials in biotechnological applications [14]. In this work, these *Alteromonas* sp. EPS were used to prepare biodegradable films, which were then characterised in terms of their optical, morphological, mechanical, and barrier properties, envisaging their potential applications in packaging, wound management, or drug delivery.

## 2. Materials and Methods

### 2.1. Exopolysaccharide Production

The EPSs were produced by six *Alteromonas* strains isolated from different locations in French Polynesia: strain Mo 169 (EPS A), Mo 278 (EPS B), and Mo 203 (EPS F) were isolated in Moorea Island lagoon; strains Fak 1576 (EPS C) and Fak 1386 (EPS E) were isolated from Fakarava atoll; and strain Tik 650 (EPS D) was isolated from Tikehau atoll [14]. All strains belong to the species *Alteromonas macleodii*, except Tik 650 that is a *Alteromonas simiduii* strain [14]. EPS production was performed by the cultivation of the *Alteromonas* strains in a 1 L fermenter, using glucose as a carbon source. The biopolymers were recovered from the cultivation broth by high-speed centrifugation (20,000× *g*, 2 h) and purified by ultrafiltration using a pellicon-2 MiniHolder equipped with a Biomax 100 K filter (Millipore Corporation, Bedford, MA, USA). Then, the supernatants were filtered through poly (ether sulphone) (PES) before ultimate concentration and lyophilised [14]. The characterisation of the purified EPS, previously reported by Concórdio-Reis et al. [14], is represented in Table 1. Briefly, the monosaccharides were analysed after either the aqueous hydrolysis or acidic methanolysis of the polymers and subsequent gas chromatography (GC) analyses as peracetylated derivatives or trimethylsilyl derivatives, respectively. For the determination of the acyl groups composition, the samples were hydrolysed with 0.1 mL 99% trifluoroacetic acid (TFA) at 120 °C, for 2 h, and analysed by liquid chromatography with an Aminex HPX-87H 300 × 7.8 mm column (Biorad, Hercules, CA, USA) coupled with a UV detector (210 nm), using sulphuric acid (H_2_SO_4_ 0.01 N) as eluent, at a flow rate of 0.6 mL/min and a temperature of 30 °C.

### 2.2. Preparation and Rheological Characterisation of Filmogenic Solutions

Aqueous solutions were prepared by dissolving the freeze-dried EPS in deionised water (1.5 wt.%) under constant stirring, at temperatures of up to 60 °C. Sodium azide (10 mg L^−1^) was added to prevent microbial growth. After dissolution, glycerol was added as a plasticiser (60 and 30 wt._glycerol_.wt._polymer_^−1^% for EPS A, and EPS B–E, respectively), and the solution was stirred for complete homogenisation. Finally, air bubbles were removed under vacuum.

The rheological characterisation of the film-forming solutions was performed on a MCR92 modular compact rheometer (Anton Paar, Graz, Austria) equipped with a cone-plate geometry (angle 2°, diameter 35 mm, 0.145 mm gap). The flow curves were obtained at 25 °C, using a steady state flow ramp in the shear rate range of 0.01 s^−1^ to 700 s^−1^. The Carreau model (Equation (1)) was employed, assuming $\eta_{\infty }$ values much lower than *η*_0_ and *η* [14,17]:

(1) $$\eta =\eta_{\infty }+\frac{\eta_{0}+\eta_{\infty }}{{\left[1+{\left(\lambda \;\dot{\gamma }\right)}^{2}\right]}^{\frac{1-n}{2}}}$$

where *η* is the apparent viscosity (Pa.s), *ɣ̇* is the shear rate (s^−1^), *λ* is a time constant (s), *η*_0_ (Pa.s) is the zero-shear rate viscosity, $\eta_{\infty }$ is the viscosity of the second Newtonian plateau (Pa.s), and *n* is the viscosity exponent.

### 2.3. Films’ Preparation

The films were obtained by casting the filmogenic solutions into polystyrene Petri dishes and left to dry at 30 °C for 48 h. Afterwards, the films were peeled from the Petri dishes and conditioned at a controlled relative humidity (RH) of 53% by placing the films inside a desiccator that contained a saturated Mg(NO_3_)_2_ solution. RH was monitored using a thermohygrometer (Vaisala, Vantaa, Finland). The films’ thickness was measured in at least six points using a digital micrometer (Mitutoyo, Andover, UK).

### 2.4. Optical Characterisation

The films’ optical and UV barrier properties were evaluated by measuring the absorbance spectrum (200–600 nm) using a CamSpec M509T spectrophotometer (Lutterworth, UK). In addition, the films’ transparency was determined as the ratio between absorbance at 600 nm (Abs_600_) and film thickness (mm) and was expressed as Abs_600_ mm^−1^ [18]. The alteration of colour on objects due to the application of the films was assessed by measuring the colour parameters of coloured paper, with and without the films. A Minolta CR-400 (Konica Minolta, Tokyo, Japan) colorimeter was used, and the CIEL*a*b* colour space was applied with the calculation of colour differences (Δ*E_ab_*), chroma (*C_ab_*), and hue (*h_ab_*), using the following equations:

(2) ΔEab=[(ΔL*)2+(Δa*)2+(Δb*)2]12

(3) Cab=(a*2+b*2)12

(4) $$h_{ab}=arctan\;\frac{b^{*}}{a^{*}}$$

where L* is the lightness, a* defines the red/green value, and b* the yellow/blue value.

### 2.5. Morphological Characterisation

The morphology of the EPS films was assessed by scanning electron microscopy (SEM) using a SEM Hitachi TM 3030Plus Tabletop. The samples were mounted on a SEM stub and coated with a thin layer of Iridium.

### 2.6. Mechanical Properties

Tensile tests were performed using a texture analyser (Food Technology Corporation, Braintree, UK) equipped with a 250 N loading cell. The samples were cut into rectangular shape pieces (15 mm × 50 mm), attached on tensile grips, and stretched at 0.5 mm s^−1^ in tension mode until break. Young’s modulus (εm, MPa) was determined within the elastic deformation of the stress–strain curve, as the initial slope of stress as a function of strain. The tensile strength at break (Ꞇ, MPa) was calculated as the ratio of the maximum force to the films’ initial cross-sectional area. The elongation at break (ε, %) was determined as the ratio of the extension of the sample upon rupture by its initial length. Five replicates of each sample were analysed.

### 2.7. Water Vapour Permeability

For the water vapour permeability (WVP) tests, the films were sealed on the top of a glass cell, with a diameter of 35 mm, which contained a saturated NaCl solution (RH = 75.3%). The cells were placed inside a desiccator that contained a saturated MgCl_2_ solution (RH = 34.7%). The desiccator was equipped with a fan to promote air circulation, and the temperature and RH inside the desiccator were measured throughout the experiment using a thermohygrometer (Vaisala, Finland). The water vapor flux was measured by weighing the cell at regular time intervals over a period of 8 h, and the WVP (mol m s^−1^ Pa^−1^) was calculated by the following equation:

(5) $$\mathrm{WVP}=\frac{N_{w}\times \delta }{\Delta P_{w.eff}}$$

where *N_w_* is the water vapour molar flux (mol m^−2^ s^−1^), δ (m) is the film thickness and Δ*P_w,eff_* (Pa) is the effective driving force, calculated as the water vapour pressure difference between both sides of the film [1]. Two film replicas were analysed for each EPS.

### 2.8. Gas Permeability

The gas permeability measurements were performed using a stainless-steel cell with two identical chambers separated by the film. The permeability was evaluated by pressurising the feed chamber up to 0.7 bar with pure gas, namely carbon dioxide (99.998%) or oxygen (99.999%) (Praxair, Maia, Portugal), followed by the measurement of the pressure change in both chambers over time, using pressure transducers (JUMO, Model 404327, Fulda, Germany). The temperature was maintained at 30 °C using a thermostatic bath (Julabo, Model EH, Seelbach, Germany). The permeability was calculated using Equation (6):

(6) $$\frac{1}{\beta }\left(\frac{\Delta p0}{\Delta p}\right)=P\;\frac{t}{\delta }$$

where Δ*p* (bar) is the pressure difference between the feed and permeate compartment, *P* (m^−2^ s^−1^, where 1 barrer = 1 × 10^−10^ cm^3^ (STP) cm cm^−2^ s^−1^ cm Hg^−1^ = 8.3 × 10^−13^ m^2^ s^−1^) is the gas permeability, t (s) is the time, *δ* (m) is the film thickness, and *β* (m^−1^) is the geometric parameter of the cell, as described by Ferreira et al. [2].

## 3. Results

### 3.1. Rheology of Filmogenic Solutions

All filmogenic solutions had the same EPS concentration (1.5 wt.%) and glycerol was added as plasticiser. Glycerol, a low molecular weight molecule, acts as a plasticiser since its presence weakens the intermolecular interactions between the polysaccharide chains, resulting in a less compact structure with enhanced flexibility [19,20]. Moreover, glycerol is also an effective humectant that can retain moisture, improving the films’ plasticity [6,12]. For the preparation of the films, glycerol was added as a plasticiser agent at a concentration of 30 wt._glycerol_.wt._pol._^−1^%. However, the film obtained with EPS A was brittle, fragile and easily breakable, making it very difficult to handle. This result is characteristic of polysaccharide films where no plasticiser was added, or the proportion of plasticiser:polysaccharide was not sufficient [12,21]. Therefore, a higher plasticiser concentration was used (60 wt._glycerol_ wt._pol._^−1^%) for this EPS, enabling the production of films that could be easily handled without breaking.

**Figure 1:**
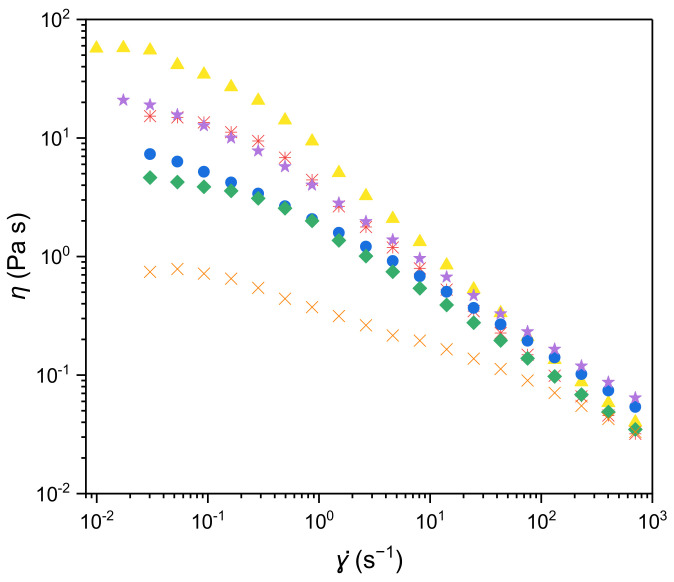
Apparent viscosity (*η*) as a function of shear rate (*ɣ̇*) for the filmogenic solutions prepared with EPS A (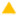), B (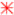), C (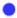), D (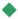), E (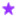), and F (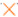), produced by *Alteromonas* strains isolated from French Polynesia (1.5 wt.% EPS, 30 wt.% glycerol for all solutions, except EPS A that was prepared with 60 wt.% glycerol).

As illustrated in Figure 1, all the filmogenic solutions prepared with the *Alteromonas* sp. EPS presented a non-Newtonian shear thinning behaviour characteristic of most polysaccharides’ solutions, in accordance with a previous rheological evaluation of these EPS solutions [14]. Additionally, the filmogenic solutions immediately recovered their original apparent viscosity upon the elimination of the shear force, resulting in overlapping flow curves for all EPS samples. A rapid recoverability is a desired feature, as it prevents dripping and guarantees films’ homogeneity after the application procedure.

The obtained flow curves were fitted to the Carreau model and the estimated parameters are presented in Table 2. For an EPS A solution, even though the EPS concentration was higher in this work (1.5 wt.%) when compared to 1 wt.% EPS studied by Concórdio-Reis et al. [14], a lower *η*_0_ value (59.2 Pa.s) was observed, in comparison with that previously reported (76.2 Pa.s). In addition, regarding the filmogenic solutions prepared with EPS B, C, and E, their *η*_0_ values were similar to those previously reported for 1 wt.% EPS (16.4 and 16.1 Pa.s, 8.06 and 11.3 Pa.s, 21.9, and 26.3 Pa.s, respectively), despite an expected increase due to the higher EPS concentration used in the present work (1.5 compared with 1 wt.%) [12]. These facts may be attributed to the presence of glycerol (60 wt.% polymer basis for EPS A and 30 wt.% polymer basis for EPSs B, C, and E) acting as a lubricant and decreasing polymer–polymer interactions. On the other hand, the *η*_0_ values of EPS D and EPS F filmogenic solutions increased despite the addition of glycerol (from 0.74 to 4.40 Pa.s, and 0.09 to 0.79 Pa.s, respectively). Differences in the molecular structure between the various EPSs is a possible explanation for the diverse behaviour of the filmogenic solutions viscosity when increasing polymer concentration in the presence of glycerol. Decreasing the filmogenic solutions’ viscosity is advantageous since it facilitates the homogenisation of the filmogenic solutions and the escape of air bubbles, which would result in defects in the film matrix (e.g., presence of pores, holes, or differences in thickness).

The rheology of filmogenic solutions is an important factor for the production of films by casting and drying at an industrial scale, since it strongly affects the type of equipment to be used and the processing conditions [19]. This information is relevant not only to produce stand-alone films, but also for the application of coatings into products by dipping, brushing, or spraying [19]. The shear thinning behaviour of EPS filmogenic solutions and the recovery of the original apparent viscosity upon the elimination of the shear force envisage suitable properties to produce films by casting using automatic film application on a flat support.

### 3.2. Film Appearance, Morphology, and Optical Characterisation

**Figure 2:**
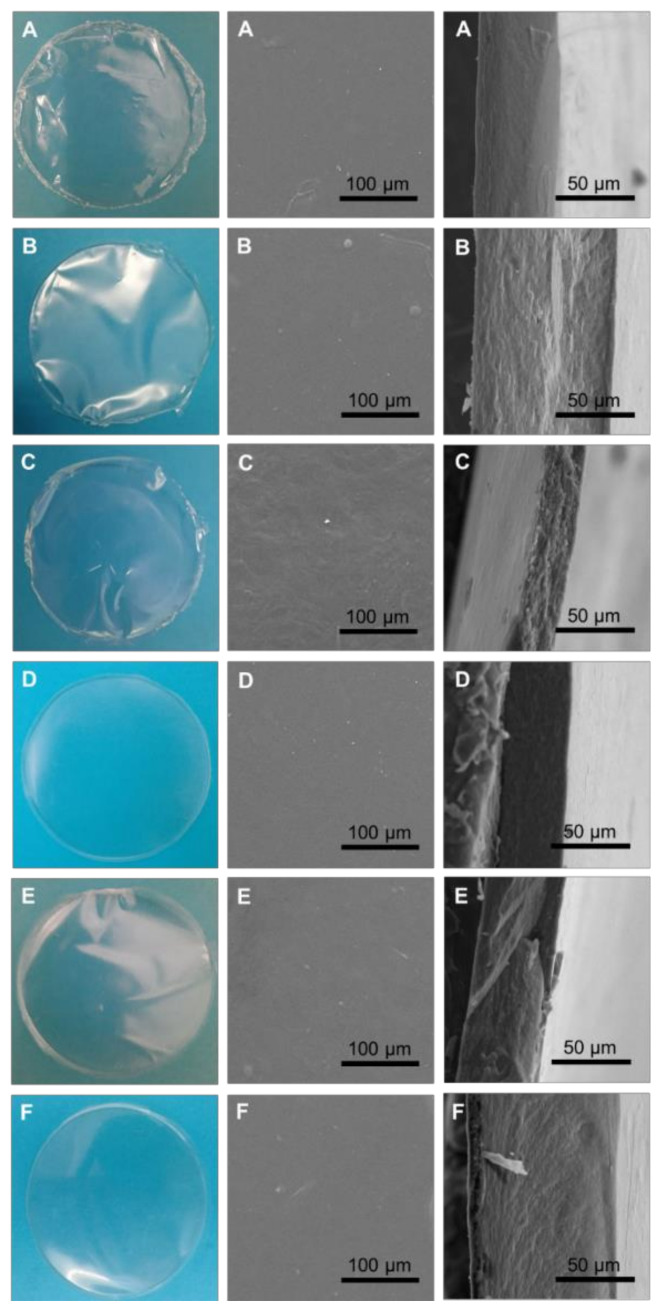
Photographs of the films prepared with *Alteromonas* EPSs (**A**–**F**) (left panel) and scanning electron microscopy images: top view (centre panel) and cross-section view (right panel).

As can be seen in Figure 2 (left panel), all the *Alteromonas* sp. EPSs formed homogeneous transparent films that were flexible but resistant when handled. Except for film C that appeared to have a rougher surface, the SEM images showed that all the films had a smooth and homogenous surface, without pores or holes (Figure 2, centre panel). The cross-section images confirmed the roughness of EPS C film and showed that the remaining films prepared with EPSs A–F had a more compact and homogenous structure with no porosity inside the membrane (Figure 2).

**Figure 3:**
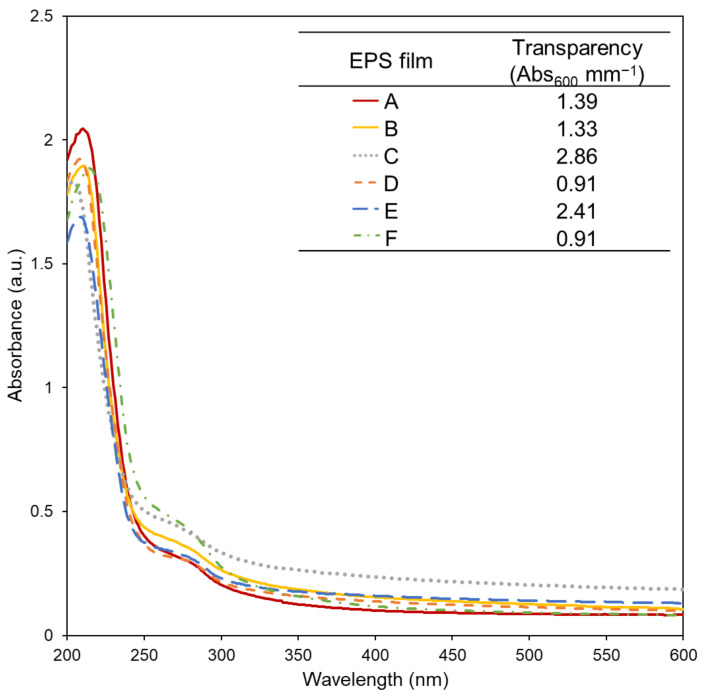
UV–vis absorption spectra and transparency (insert) of the films prepared with EPSs (**A**–**F**) produced by *Alteromonas* strains isolated from French Polynesia.

As presented in Figure 3, no significant optical bands were found in the spectra from 400 to 600 nm, thus showing that the films were transparent. In this range, the optical density of the films prepared with EPSs C and E were higher, suggesting that these films were opaquer (Figure 3). Interestingly, EPSs C and E presented the lowest uronic acid (17 and 22 mol%, respectively, compared with 38–46 mol%) and acyl contents (1.1 and 1.0 wt.%, respectively, compared with 2.1–6.2 wt.%) (Table 1). Nonetheless, the films’ transparency values were within the range of those reported for other biopolymer films, namely chitosan (1.9) [12], gelatine (0.67) [2], and Kefir microflora EPS (2.71) [19]; and lower than those reported for cassava starch (4.7), corn starch (4.6) [18], and *Enterobacter* A47 EPS films (3.67) [2]. Commonly used synthetic plastics films, such as low-density polyethylene (LDPE), oriented polypropylene and polyvinyl dichloride, also presented similar transparency values (3.05, 1.67, and 4.58, respectively) [18,19]. Moreover, the absorption band observed between 200 and 300 nm suggests that the EPS films show barrier properties to UV radiation [18] (Figure 3) which might be further improved by increasing the thickness of the films.

**Figure 4:**
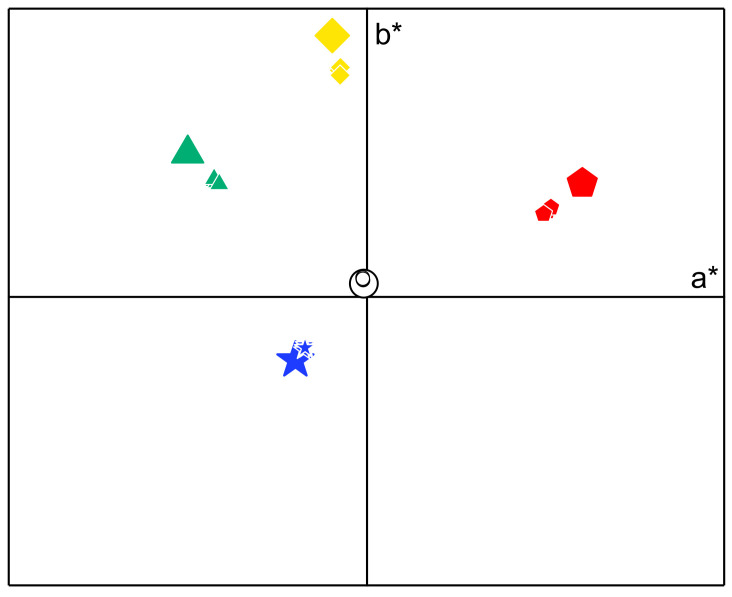
Parameters a* and b* of the CIELAB system for coloured paper sheets uncovered (large symbols) and covered (small symbols) by the EPS films.

The colour changes in the objects were investigated by comparing the colour parameters of coloured paper sheets uncovered and covered by the films. The CIEL*a*b* colour parameters for five different colours are presented in Figure 4 and Table 3. For all EPS films, the hue (h*_ab_, angle towards the horizontal axes) did not change with the application of the film (Figure 4). However, there was an approximation towards the origin, indicating that the colour saturation (chroma, C*_ab_) decreased (Figure 4). Considering the colour alteration (Δ*E_ab_*) (Table 3), for all *Alteromonas* sp. EPS films, the values were low (1.7–12.6), and the lowest changes were observed for white and blue colours (Δ*E_ab_* < 5). Even low, these alterations may be perceived by the human eye, since colour differences with Δ*E_ab_* values below 1.5 are classified as small, and only Δ*E_ab_* values below 0.2 are not perceptible [22].

Higher Δ*E_ab_* values were found for the films prepared with *Enterobacter* A47 EPS in all colours (9.2–19.8) [12]. Chitosan films also presented higher Δ*E_ab_* values in blue and white coloured surfaces (11.6 and 9.7, respectively), but presented similar values for the other colours tested (9.9–12.5) [12].

The optical properties, namely the transparency and UV light absorption capacity of polysaccharide films, are relevant for some of their applications. For example, for food packaging, transparency is an important feature for consumer acceptability, while UV barrier properties are useful to prevent the oxidative degradation of fatty foods, thus increasing the shelf life of food products [18]. Moreover, for medical applications, UV barrier properties could be advantageous for skin therapeutics (e.g., in transdermal drug delivery systems), and transparency is useful to control the wound healing process without the need to remove the membrane, or for bioactive coatings of products and devices [23].

### 3.3. Mechanical Properties

Mechanical characterisation is essential for the application of the films as biomaterials, for example, in wound dressings, synthetic tissues, or food packaging [6,13,21,24]. Therefore, the films prepared with *Alteromonas* EPSs were subjected to tensile tests and the obtained mechanical parameters are presented in Table 4. It is noticed that the EPS films displayed different mechanical behaviours. EPSs A, B, and C presented typical mechanical characteristics of flexible films, with lower stiffness, showed by the lower tensile strength (Ꞇ) (4.55–11.7 MPa), higher elongation at break (ε) (35.6–47.0%), and lower Young’s modulus (εm) (10–93 MPa). In contrast, EPSs D, E, and F seemed to form more rigid films, characterised by higher Ꞇ values (16.6–23.6 MPa), lower ε (2.80–5.58%) and higher εm (597–1100 MPa) (Table 4). Interestingly, the Ꞇ of these films is higher to that of LDPE (0.9–14 MPa), poly(ε-caprolactone) (14 MPa), and polyvinyl chloride (PVC, 15 MPa) films [3,25]. These differences in mechanical behaviour between *Alteromonas* sp. EPS films could not be correlated with their differences in chemical composition or molecular weight (Table 1). Nonetheless, the EPSs might present variations in their polymer chain structures that impact the inter-chain interactions and, therefore, their mechanical characteristics [26].

The large range of variables involved in the mechanical behaviour of films might compromise the comparison between polymer films, since not only the mechanical characteristics are strongly influenced by the factors stated above, but also by the film preparation techniques (e.g., type and concentration of the plasticiser) and conditioning conditions (e.g., RH values) [2,19,21]. Despite this, both the Ꞇ and ε values (4.55–23.6 MPa and 2.80–47.0%, respectively) are within those found for several polysaccharide-based films (3.1–75.1 MPa and 2.5–116.7%, respectively) (Table 4). Of notice were the high ε values determined for the films prepared with EPSs A, B, and C (Table 4), which were superior to those reported for several commercialised films such as polystyrene (2–3%), poly(3-hydroxybutyrate) (5–8%), cellophane (14.4%), and poly(L-lactic acid) (9%) [3,27]. Such high ε values suggest that those EPS films have potential for applications where a high elasticity is required, such as for wound dressings, since the high flexibility allows the films to easily adapt to the wound shape and deform according to the skin movements [13]. Moreover, the higher ε values found for the *Enterobacter* A47 EPS film prepared with citric acid (50%) as plasticiser [2] and the increase in ε values found for Kefir microflora EPS films prepared with increasing glycerol concentrations [19] seem to indicate that the plasticity of the *Alteromonas* sp. films might be optimised by adding more glycerol or by using a different plasticiser.

Additionally, the tensile strength of the EPS films might be improved by blending the EPSs with other biopolymers (e.g., gelatine, chitosan, or bacterial cellulose) [28] or by the addition of reinforcing materials (e.g., starch nanocrystals, nano/microfibrils of bacterial cellulose or chitin whiskers) [21]. For example, the *Nostoc commune* colonies EPS films were successfully reinforced by the addition of starch nanoparticles and chitin whiskers [29].

**Table 4:** Mechanical properties, water vapour permeability (WVP), and gas barrier properties of films prepared with the *Alteromonas* EPSs and of other carbohydrate-based films reported in the literature (Ꞇ, Tensile Strength at Break; ε, Elongation at Break; εm, Young’s Modulus; Gly, glycerol; RH, relative humidity; CMC, carboxymethyl cellulose; n.a., not available; n.d. not detected).

Film Composition	Mechanical Properties			Plasticiser (wt. wt._polymer_^−1^% or g L^−1^)	RH (%)	WVP (10^−11^ mol m^−1^ s^−1^ Pa^−1^)	Driving Force (ΔRH%)	Permeability (10^−16^ mol m^−1^ s^−1^ Pa^−1^)		Reference
	Ꞇ (MPa)	ε (%)	εm (MPa)					O_2_	CO_2_	
EPS A	4.55 ± 0.36	47.0 ± 1.1	10 ± 0	Gly (60% or 9 g L^−1^)	53	5.8 ± 0.7	75.3–34.7	43.1 ± 10.1	62.6 ± 11.0	This study
EPS B	11.7 ± 1.1	37.7 ± 0.5	93 ± 12	Gly (30% or 4.5 g L^−1^)		2.7 ± 0.1		7.5 ± 0.4	24.0 ± 1.2	This study
EPS C	10.8 ± 1.1	35.6 ± 5.5	65 ± 5			6.1 ± 1.0		30.0 ± 1.5	28.5 ± 1.4	This study
EPS D	23.6 ± 2.8	5.58 ± 0.83	1100 ± 110			3.7 ± 0.4		n.d.	n.d.	This study
EPS E	21.1 ± 0.1	4.40 ± 0.42	597 ± 62			2.9 ± 0.0		n.d.	n.d.	This study
EPS F	16.6 ± 0.4	2.80 ± 0.46	885 ± 125			5.8 ± 0.2		n.d.	n.d.	This study
*Enterobacter* A47 EPS	3.8–15.5	5.4–22.1	14.5–457.8	Gly (30%)	45	1.7–2.3	80.9–53.4	n.a.	n.a.	[26]
	3.1	54.9	2.8	Citric acid (50%)	44.3	1.0	76.9–22.5	0.7	42.7	[2]
*Pseudomonas oleovorans* EPS	51	9.5	1738	None	44.3	1.1	64.8–22.0	n.a.	2.0	[1]
						5.4	92.0–64.8			
Kefir microflora EPS	40.9	2.70	n.a.	None	75	0.32	75–0	n.a.	n.a.	[19]
	15.2	116.7	n.a.	Gly (25%)		0.23				
Chitosan	31.1	10.6	n.a.	None	n.a.	n.a.	-	n.a.	n.a.	[20]
	41.6	24.7	1193	Gly (15%)	n.a.	n.a.	-	n.a.	n.a.	[16]
Chitosan/dextran-like EPS	43.3	20.7	n.a.	1,3-propanediol (50%)	n.a.	n.a.	-	n.a.	n.a.	[20]
Chitosan/ *Alteromonas* sp. EPS	39.5–42.7	16.6–23.7	1008–1186	Gly (15%)	n.a.	n.a.	-	n.a.	n.a.	[16]
Gellan	30	34	n.a.	Gly (4%)	54	2.0	54–0	n.a.	n.a.	[30]
Alginate crosslinked with calcium	64.7	2.8	n.a.	Gly (40%)	56	n.a.	-	n.a.	n.a.	[31]
	24.1	7.6	n.a.		98	2.6	100–0	n.a.	n.a.	
	65.9	2.5	n.a.	Sorbitol (40%)	56	n.a.	-	n.a.	n.a.	
	18.4	6.6	n.a.		98	1.7	100–0	n.a.	n.a.	
Pectin/alginate/xanthan	29.7	19.0	n.a.	Gly (18 g L^−1^)	50	1.01	100–0	n.a.	n.a.	[25]
Hyaluronic acid (HA)	70.7	5.6	n.a.	none	58	n.a.	-	n.a.	n.a.	[32]
HA/CMC	68.8–75.1	7.9–13.6	n.a.	none	58	n.a.	-	n.a.	n.a.	
Cassava starch/CMC	13.3	65.7	n.a.	Gly (15 g L^−1^)	55	0.92	100–0	n.a.	n.a.	[6]

### 3.4. Water Vapour Permeability

WVP is a crucial factor for the application of films and is dependent on several factors, including hydrophobicity, diffusion rate, solubility coefficient, crystalline, amorphous regions ratio, film density, and polymer chain mobility [6]. As presented in Table 4, all *Alteromonas* sp. EPS films had high WVP values (2.7–6.1 × 10^−11^ mol m^−1^ s^−1^ Pa^−1^) for a driving force of 75.3–34.7% RH. These values were higher than those found for other polysaccharide films (1.0–2.3 × 10^−11^ mol m^−1^ s^−1^ Pa^−1^) tested for similar driving forces (Table 4). It is known that WVP is a very important property for wound management applications. Providing the accurate moist environment for correct healing is a major feature demonstrated by some commercially available dressings, such as Tegaderm and OpSite [33]. Moreover, the transparency observed in *Alteromonas* sp. EPS films allows easy wound monitorisation which suggests that these films might have a potential in this field [23,33]. On the contrary, low WVP values are preferable for food packaging in order to maintain the moisture content of the food [25]. For example, synthetic polymers used as packaging materials, such as LDPE and polyethylene terephthalate (PET), both had a WVP of 0.01 × 10^−11^ mol m^−1^ s^−1^ Pa^−1^ for a driving force of 90–0% RH [34], and cellophane that presented a WVP of 0.47 × 10^−11^ mol m^−1^ s^−1^ Pa^−1^ with 72–0% RH [27]. Nonetheless, different strategies could be employed to increase the films barrier properties to water vapor, such as promoting crosslinking reactions between the polymer’s chains, or the addition of lipids (e.g., olive oil, rice wax, or beeswax) to the filmogenic solutions or in multilayer films [35].

### 3.5. Gas Permeability

For transdermal drug delivery, oxygen barrier properties might be important in maintaining the stability of the entrapped bioactive molecules [7]. Polysaccharides films are effective gas barriers at a low RH due to their hydrogen-bonded dense polymer matrix [12]. However, it significantly increases for increasing ambient moisture due to the plasticising effect of water [12]. *Alteromonas* sp. EPS A film presented the highest permeability value for both gases (43.1 ± 10.1 and 62.6 ± 11.0 × 10^−16^ mol m^−1^ s^−1^ Pa^−1^ for O_2_ and CO_2_, respectively), followed by the films prepared with EPS C (30.0 ± 1.5 and 28.5 ± 1.4 × 10^−16^ mol m^−1^ s^−1^ Pa^−1^ for O_2_ and CO_2_, respectively) and EPS B (7.5 ± 0.4 and 24.0 ± 1.2 × 10^−16^ mol m^−1^ s^−1^ Pa^−1^ for O_2_ and CO_2_, respectively). The permeability of an EPS A film was also superior to other polysaccharide films such as those prepared with FucoPol, chitosan, and galactomannans (0.13–2.3 and 14.7–42.7 × 10^−16^ mol m^−1^ s^−1^ Pa^−1^ for O_2_ and CO_2_, respectively) under similar RH conditions (32.4–50%) [2]. Permeability to gases is a main issue in wound management. Being able to provide the correct exposure of the wound to oxygen or carbon dioxide can facilitate cell regeneration and protect against the development of dangerous microbial growth, therefore enhancing the healing process [4]. High gas permeability is a major feature of commonly used dressings such as Hyalosafe and Hydrofilm [33].

For EPSs D, E, and F it was not possible to measure the gas permeability since no pressure alterations were found after 48 h, suggesting that these films are excellent barriers to oxygen and carbon dioxide. For food packaging applications, low gas permeation to oxygen or carbon dioxide is very important for food preservation [36]. In fact, these EPS had better gas barrier properties compared with synthetic polymers usually used as packaging materials, such as LDPE (10.03 and 42.2 × 10^−16^ mol m^−1^ s^−1^ Pa^−1^ for O_2_ and CO_2_, respectively) or PET (0.12 and 0.38 × 10^−16^ mol m^−1^ s^−1^ Pa^−1^ for O_2_ and CO_2_, respectively) [2].

## 4. Conclusions

Six *Alteromonas* sp. exopolysaccharides were investigated for the preparation of biodegradable films by casting, using glycerol as a plasticising agent. The films were transparent, and their application caused small colour alterations when applied over coloured surfaces. Considering their mechanical properties, two distinct behaviours were found: three EPSs formed flexible films, with high elongation at break and low tensile strength at break, whereas the other three EPS films were stiffer and more resistant, with low elongation at break, and high tensile strength at break. The higher elongation values described herein are superior to those found in the literature for other EPS films prepared with the same content in glycerol, suggesting their potential for applications that require flexible films. EPSs A, B, and C presented high carbon dioxide and oxygen permeabilities, whereas EPSs D, E, and F proved to be excellent gas barriers. In addition, the films had high water vapour permeabilities due to their hydrophilic character. These features are very important for wound management applications since it is of great importance to maintain water balance and good contact in the wound-dressing interface with the possibility to absorb wound exudate. Given this, the flexible transparent and permeable films produced using EPSs A, B, and C revealed to be great candidates for the development of dressing materials to cover and protect wounds, while monitoring the wound healing process. On the other hand, EPS D, E, and F, by being stiffer and due to their great barrier properties, could be used in food packaging applications alone or as one layer of multilayer polymeric films. Nonetheless, different strategies, such as changing the type and concentration of plasticiser, the incorporation of additives or blending the EPSs with other biopolymers, must be studied to meet the requirements for those applications.

## Figures and Tables

**Table 1:** Characterisation of the EPSs produced by *Alteromonas* strains isolated from French Polynesia marine environments: carbohydrate (Fuc, fucose; Gal, galactose; GalA, galacturonic acid; Glc, glucose; GluA, glucuronic acid; Man, mannose; Rha, rhamnose) and acyl groups (Ac, acetate; Pyr, pyruvate; Suc, succinate) composition; sulphate, uronic acids, protein, and inorganic salts content; average molecular weight (Mw); polydispersity index (PDI); and thermal degradation temperature (T_deg_). Reproduced from Concórdio-Reis et al. [14].

EPS	Monosaccharide Composition (Molar Ratio)	Acyl Groups (wt%)	Sulphate (wt%)	Protein (wt%)	Inorganic Content (wt%)	M_w_ (MDa)	PDI	T_deg_ (°C)
A	Glc:GlcA:Man:Gal:GalA (2.5:2.5:1.5:1.5:1.5)	Ac (0.5 ± 0.1) Pyr (4.9 ± 0.1)	2.8 ± 0.1	2.8 ± 0.4	7.7 ± 1.5	1.6 4.6 ^1^	1.3 1.3 ^1^	269
B	Gal:GlcA:Glc:GalA (2:1.5:1:1)	Ac (0.5 ± 0.0) Pyr (0.2 ± 0.0) Suc (1.8 ± 0.1)	3.3 ± 0.3	3.5 ± 0.1	20.0 ± 1.7	4.6	1.4	265
C	Gal:Rha:Man:Glc:GlcA:GalA:Fuc (4.5:2:1.5:1:1:1:0.5)	Pyr (1.1 ± 0.2)	3.4 ±0.0	2.6 ± 1.0	34.7 ± 0.2	1.2	1.4	268
D	GlcA:Glc:Gal:Man:GalA (3:2:2:1.5:1.5)	Ac (2.1 ± 0.0)	2.0 ± 0.0	6.8 ± 0.2	11.4 ± 0.3	1.4	1.5	262
E	Gal:Man:Rha:Glc:GlcA:GalA (3:2:1:1:1:1)	Suc (1.0 ± 0.0)	2.9 ± 0.0	2.1 ± 0.3	15.0 ± 0.1	2.5 4.3 ^1^	1.1 1.5 ^1^	267
F	Glc:Gal:GlcA:Rha:GalA (2:2:2:1:1)	Ac (0.7 ± 0.0) Pyr (5.5 ± 0.3)	3.4 ± 0.0	1.7 ± 0.0	13.7 ± 0.3	3.2	1.2	260

^1^ EPS presented two distinct peaks in the size exclusion chromatography plots.

**Table 2:** Carreau model parameters (*η*_0,_ zero-shear rate viscosity; *n*, viscosity exponent; *λ*, time constant) estimated for the filmogenic solutions prepared with the different EPSs produced by *Alteromonas* strains isolated from French Polynesia.

EPS	*η*_0_ (Pa.s)	*n* (-)	*λ* (s)	r^2^
A	59.2 ± 1.1	0.374 ± 0.036	22.6 ± 2.5	0.996
B	16.4 ± 0.3	0.329 ± 0.050	8.30 ± 1.00	0.995
C	8.06 ± 0.15	0.549 ± 0.008	25.3 ± 1.9	0.999
D	4.40 ± 0.08	0.474 ± 0.028	5.95 ± 0.69	0.996
E	21.9 ± 0.3	0.463 ± 0.014	27.2 ± 1.9	0.999
F	0.79 ± 0.01	0.667 ± 0.010	9.79 ± 1.04	0.998

**Table 3:** Parameters a*, b*, and L* of the CIELAB system and calculated colour alteration (Δ*E_ab_*) that resulted from the covering of coloured paper sheets with the different EPS films.

Colour	Parameter	EPS					
		A	B	C	D	E	F
White	a*	−0.80 ± 0.01	−0.72 ± 0.02	−0.80 ± 0.01	−0.86 ± 0.05	−0.84 ± 0.01	−0.89 ± 0.07
	b*	5.4 ± 0.1	4.6 ± 0.0	5.0 ± 0.0	5.0 ± 0.5	4.8 ± 0.1	5.4 ± 0.4
	L*	92.8 ± 0.1	93.1 ± 0.1	93.3 ± 0.1	93.4 ± 0.4	93.4 ± 0.0	92.9 ± 0.5
	Δ*E_ab_*	2.5 ± 0.1	1.8 ± 0.1	1.9 ± 0.0	1.8 ± 0.7	1.7 ± 0.0	2.4 ± 0.7
Green	a*	−34.1 ± 0.0	−33.9 ± 0.1	−32.9 ± 0.3	−33.8 ± 0.2	−33.4 ± 0.0	−33.7 ± 0.2
	b*	32.7 ± 0.0	32.2 ± 0.0	31.3 ± 0.3	32.1 ± 0.2	31.9 ± 0.1	32.3 ± 0.1
	L*	69.2 ± 0.0	69.7 ± 0.0	70.1 ± 0.1	69.9 ± 0.1	70.2 ± 0.0	69.4 ± 0.1
	Δ*E_ab_*	9.1 ± 0.0	9.6 ± 0.1	11.0 ± 0.4	9.8 ± 0.3	10.3 ± 0.1	9.7 ± 0.2
Blue	a*	−14.1 ± 0.1	−13.5 ± 0.1	−13.7 ± 0.0	−13.9 ± 0.0	−13.5 ± 0.1	−13.7 ± 0.1
	b*	−14.8 ± 0.3	−14.4 ± 0.1	−14.1 ± 0.1	−14.2 ± 0.0	−14.0 ± 0.1	−13.3 ± 0.1
	L*	58.7 ± 0.0	58.9 ± 0.0	58.7 ± 0.2	58.2 ± 0.3	59.2 ± 0.4	57.7 ± 0.4
	Δ*E_ab_*	3.3 ± 0.3	4.0 ± 0.1	4.1 ± 0.1	3.9 ± 0.0	4.4 ± 0.2	4.8 ± 0.1
Yellow	a*	−5.8 ± 0.1	−6.1 ± 0.0	−5.9 ± 0.0	−6.3 ± 0.1	−6.1 ± 0.0	−6.0 ± 0.2
	b*	63.7 ± 0.0	63.1 ± 0.0	61.5 ± 0.1	63.5 ± 0.2	62.9 ± 0.1	63.3 ± 0.5
	L*	88.4 ± 0.2	89.0 ± 0.1	89.0 ± 0.1	89.2 ± 0.1	89.3 ± 0.0	88.7 ± 0.2
	Δ*E_ab_*	9.2 ± 0.1	9.6 ± 0.0	11.2 ± 0.1	9.2 ± 0.2	9.8 ± 0.1	9.5 ± 0.5
Red	a*	41.3 ± 0.2	40.7 ± 0.1	39.6 ± 0.1	40.9 ± 0.6	40.5 ± 0.2	40.7 ± 0.1
	b*	24.8 ± 0.1	24.1 ± 0.0	23.1 ± 0.1	24.0 ± 0.3	23.8 ± 0.1	24.4 ± 0.2
	L*	59.3 ± 0.0	59.9 ± 0.0	60.5 ± 0.0	60.7 ± 0.8	60.4 ± 0.1	59.7 ± 0.2
	Δ*E_ab_*	10.0 ± 0.2	11.0 ± 0.1	12.6 ± 0.2	11.2 ± 1.1	11.5 ± 0.2	10.8 ± 0.3

## Data Availability

The data presented in this study are available on request from the corresponding author.
